# Cone-Beam Computed Tomography Analysis on the Relationship between the Mandibular Third Molar and the Position of the Mandibular Canal in Koreans from the Yanbian Area and the Han People

**DOI:** 10.1155/2023/9563476

**Published:** 2023-01-03

**Authors:** Zhen-Long Liu, En-Shi Jiang, Lian-Yan Cui, Jing-Xu Li

**Affiliations:** Department of Stomatology, Affiliated Hospital of Yanbian University, Yanji 133000, China

## Abstract

**Objective:**

To analyze differences in the positional relationships between the mandibular third molar (MTM) and the mandibular canal in Korean and Han patients using cone-beam computed tomography (CBCT) and to provide a basis for preoperative risk assessments.

**Materials and Methods:**

The CBCT imaging data of 260 Korean and Han patients were collected. The patients' genders, ages, impaction types and depths, relative positions between the MTMs and the mandibular nerve canals, and the shortest distances and shapes at the root tips and cortical bones were all recorded and analyzed. All data were compared using the nonparametric test, ordered logistic regression analysis, a chi-square test, and Fisher's exact test.

**Results:**

The relationship between the mandibular canal and the relative position of the MTM differed between Korean and Han patients, mainly in the different types of impactions, and the difference was statistically significant (*P* < 0.05). The shortest distance between the mesioangular and horizontally impacted mandibular canals and the buccal side of the MTM in Korean patients was less than in Han patients, and the difference was statistically significant (*P* < 0.05). For horizontal impactions, the probability of cortical bone interruption was 1.980 times greater in Korean patients than in Han patients, and the difference was statistically significant (*P* < 0.05). The significance threshold was set at 0.05.

**Conclusion:**

There are some differences in the positional relationship between the mandibular canal in the MTM region and the rate of cortical bone disruption between Koreans from the Yanbian area and the Hans. This should gain clinical attention.

## 1. Introduction

Impacted mandibular third molars (MTMs) may lead to caries, root resorptions, periodontal bone loss, cysts, or tumor formations [1]. Extracting the impacted MTM is a common surgical treatment in clinics, but one of its most serious complications is inferior alveolar nerve (IAN) injury. The incidence of IAN injury in MTM extractions is 0.4%–8% [2]. The MTM root, being adjacent to the mandibular canal, is the most prominent risk factor for IAN injury [3].

Bell et al. [4] proved that the diagnostic accuracy of panoramic radiography is relatively poor, so they recommended cone-beam computed tomography (CBCT) because it can provide accurate morphological images [5]. This includes the buccolingual structure, root number and curvature, shape of the mandibular canal, cortical disruption, and the distance between the mandibular canal and the MTM [6–8]. According to a recent study [9], the incidence of IAN injury is 28.6% when the mandibular nerve canal is located on the lingual side of the MTM, 28% when the mandibular nerve canal is in contact with the MTM, and 11.7% when the mandibular nerve canal is in contact with the MTM. This is significantly higher than the incidence of IAN injury during MTM extraction. Therefore, the use of CBCT can clarify the position of the mandibular canal in relation to the MTM preoperatively, predict the possibility of IAN injury, and facilitate communication between the surgeon and the patient.

It is important to be familiar with the situation of the mandibular canal in the MTM area in various demographics. Some scholars have confirmed differences in the dental, maxillofacial, and craniofacial areas of different nationalities and in different regions [10, 11]. The mandibular plane of Korean people is flatter than that of Han people, and the mandible of Korean people shows a counterclockwise rotation and a low-angle trend [11]. The different growth and rotation modes of the mandible are related to the position and curvature of the mandibular canal [12]. There are differences in the position of the mandibular canal among different ethnic groups [13], and the rate of cortical bone disruptions is also different under different positional relationships of the mandibular canal in the MTM [7, 14]. This study aims to analyze differences in the positional relationship between the MTM and the mandibular canal between Korean people from the Yanbian area and Han people. This study also aims to fill different nationality databases and supply an imaging basis to provide a reference for future clinical preoperative risk assessment.

## 2. Materials and Methods

### 2.1. Subjects

Data of patients that underwent MTM extraction from the Department of Stomatology, Affiliated Hospital of Yanbian University, from October 2019 to May 2021 were collected. A total of 260 patients with their MTM roots contacting or overlapping with the nerve tubes were screened by panoramic radiography, and their CBCT files were collected.

#### 2.1.1. Inclusion Criteria

① Over 18 years old
② Of Korean or Han nationality
③ Had fully developed MTM roots

#### 2.1.2. Exclusion Criteria

① Did not have obvious inflammation in the apical region
② Did not have obvious inflammatory lesions, various types of developmental cysts, or tumor lesions in the MTM mandible region

### 2.2. Study Methods

Scanning equipment: the digital image data were sourced using a CBCT machine (CBCT, KaVo 3D eXam, Germany, Biberach), and the Digital Imaging and Communications in Medicine file format was used for storage. The operating parameters were 5 mA and 120 kV, with a 0.3-mm fixed focal spot and pixels set to 640 × 640.

Cone-beam computed tomography data processing software was the eXamVision cone-beamthree-dimensional imaging system.

Third-party computed tomography image measurement software was Materialise SIMPLANT Pro 17.01 (Manufacturer Materialise, head office in Flanders, Belgium).

### 2.3. Cone-Beam Computed Tomography Image Reading Analysis

**Figure 1:**
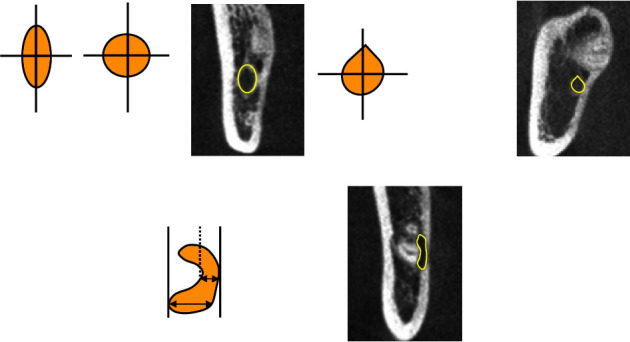
Cone-beam computed tomography (CBCT) image of the shape of the mandibular canal at the shortest distance from the mandibular third molar (MTM). The yellow arrow represents the mandibular canal. (a) Round/oval; (b) teardrop shape; (c) dumbbell shape.

**Figure 2:**
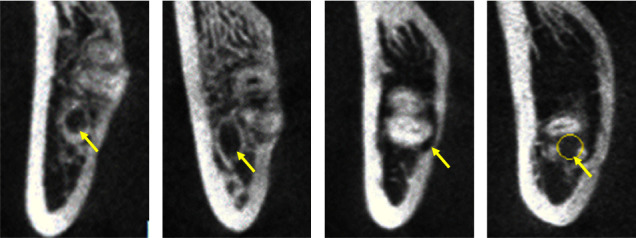
The CBCT images of the mandibular canal at different locations of impacted MTM. The yellow arrow represents the mandibular canal. The yellow arrow represents the mandibular canal. (a) Located below the root; (b) located on the buccal side; (c) on the lingual side; (d) located between roots.

**Figure 3:**
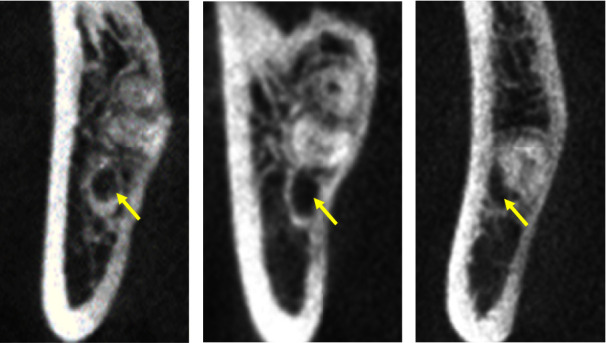
The CBCT image of the cortical bone state of the mandibular canal in the apical region of MTM. The yellow arrow represents the mandibular canal. (a) The root of the MTM and the cortical bone of the mandibular canal is intact without contact; (b) the root of the MTM is in contact with the intact cortical bone of the mandibular canal; (c) the root of the MTM is in contact with the disrupted cortical bone of the mandibular canal.

**Figure 4:**
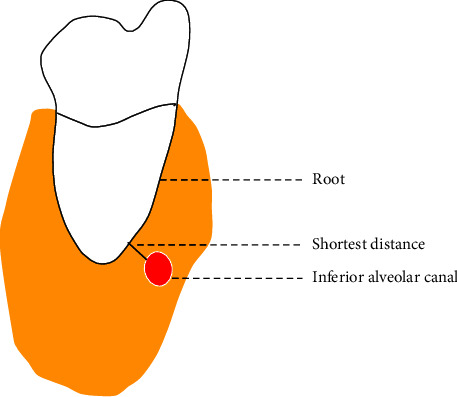
Minimum distance from the mandibular canal to the MTM root.

1. The Winter classification was used to classify the impaction angles of the MTM. The classes of impaction were vertical, mesial, horizontal, buccal, lingual impaction, distal, and inverted [15].
2. The Pell classification was used to record and divide the MTM impaction depths into high, middle, and low levels [16].
3. The Ueda classification was used to identify the cross-sectional shape of the mandibular canal when the distance between it and the MTM was at its shortest in the coronal section. The shapes were round/oval, dumbbell, or teardrop and are shown in Figure 1 [7].
4. Maegawa's classification was used as a reference to classify the positional relationship between the mandibular canal and the MTM at their shortest recorded distance in the coronal section. The MTM and mandibular canal were located below the MTM root tip, the buccal and lingual positions, and between the tooth roots and are shown in Figure 2 [17].
5. According to Park's classification [6], the cortical bone with the shortest distance between the mandibular canal and the MTM was categorized as either intact noncontact cortical bone, intact contact cortical bone, or disrupted cortical bone, as shown in Figure 3.
6. The defining principle of root shape and number was used to divide the MTM root number into single, double, three, or four roots [8].
7. The shortest distance between the mandibular canal on the coronal plane and the root tip of the MTM was measured (accurate to 0.01 mm), and the average was recorded after three measurements, as is shown in Figure 4.

### 2.4. Statistical Methods

All data were analyzed using IBM SPSS Statistics26.0 (Manufacturer International Business Machines Corporation, head office in Armonk, New York, USA) statistical software. Subject to skewed distribution, the measurement data were expressed using the median and interquartile range (P_25_–P_75_) and analyzed using a nonparametric test. The enumeration data were expressed as the sample size and percentage and compared using ordered logistic regression analysis, a chi-square test, and Fisher's exact test. The difference was considered statistically significant at *P* < 0.05.

## 3. Results

### 3.1. General Clinical Data and Distribution of Impaction Types

According to the inclusion criteria, a total of 260 patients who underwent panoramic radiography showing contact or overlap between the MTM tooth root and the mandibular canal were screened in this study. They included 130 Korean patients (67 men and 63 women) and 130 Han patients (62 men and 68 women). There was no significant gender difference in the number of Korean and Han patients (*P* > 0.05). The age of the Korean and Han patients had a skewed distribution: the median age of the Korean patients was 29.0 (23.0–35.0) years, and the median age of the Han patients was 27.0 (24.0–32.0) years. There was no significant difference between the ages (*Z* = −1.501, *P*=0.133 > 0.05), as is shown in Table 1. There were a total of 421 impacted MTMs in the 260 patients. There was no significant difference in the frequency of MTMs between the Korean and Han patients by gender, location, and type of impaction (*P* > 0.05), as is shown in Table 2.

### 3.2. Comparative Analysis of the Different Positional Relationships

The relative positional relationship between different mandibular canals and MTMs in the Korean and Han patients was compared, and the results are shown in Table 3.

The percentage of mandibular canals located on the buccal side of the MTM in the Korean patients was significantly lower than that of the Han patients, and the difference was statistically significant (*P* < 0.05). The percentage of mandibular canals located between the MTM roots in the Korean patients was significantly higher than in the Han patients, and the difference was statistically significant (*P* < 0.05).

#### 3.2.1. Analysis of the Positional Relationship and the Shortest Distance between the Mandibular Canals and Mandibular Third Molars in Different Impaction Types

The inverted, lingual, distal, and buccal impaction samples accounted for too small a sample size for each, so the vertical, mesial, and horizontal impaction samples were selected for statistical analysis. The positional relationship and the shortest distance between the root and buccal side for the vertical, mesial, and horizontal impactions of the Korean and Han patients were compared. When the mandibular canal was located between the lingual side and the root, the shortest distance sampled between the Korean and Han patients was 0, which could not be statistically analyzed.

For mesial impactions, the percentage of the mandibular canals located on the lingual side of the MTMs in the Korean patients was significantly lower than that in the Han patients, which was statistically significant (*P* < 0.05). However, the proportion of mandibular canals located between the roots of the MTMs in the Korean patients was significantly higher than in the Han patients, which was statistically significant (*P* < 0.05). In horizontal impactions, the percentage of mandibular canals located between the roots of the MTM in the Korean patients was significantly higher than that in the Han patients, which was statistically significant (*P* < 0.05), and the results are shown in Table 4.

There was a significant difference in the median buccal shortest distances between the mesial and horizontal impaction (*P* < 0.05). According to the P_25_–P_75_ comparison, the shortest distance samples of the Han and Korean patients were greater for the mesial and horizontal impactions. The comparison of the shortest distances is shown in Table 5.

### 3.3. Comparative Analysis of the Relationship between the Shape and Position of Different Mandibular Canals and the Cortical Bone

As is shown in Table 6, the results found that the shape distribution of the mandibular canal differed depending on the positional relationship (*χ*^2^ = 169.349, *P* < 0.05).

When the mandibular canal was located below the MTM root, on the buccal side, its shape in the Korean and Han patients was mainly round/oval. When the mandibular canal was located on the lingual side, between the roots of the MTM, its shape in the Korean and Han patients was mainly dumbbell-like. However, there was no significant difference in the shape of the mandibular canal in the Korean and Han patients in the same positional relationship (*P* > 0.05).

### 3.4. Comparative Analysis of the Risk of Cortical Bone Disruption with Different Impaction Types, Depths, and Root Numbers

Taking the cortical bone status as the dependent variable, the ordered logistic regression analysis method was used to study the nationality, impaction type, depth, and root number. Taking the Han patients as the reference level, the odds ratio value and its 95% confidence interval were obtained to represent the relative risk of cortical bone disruption in the Korean patients under the impaction type, depth, and root number of roots, as is shown in Table 7.

The results showed that the incidence of cortical bone disruption in the mandibular canal in the MTM area of the Korean patients was higher than in the Han patients. The results also demonstrated that there was no significant difference in the risk of cortical bone disruption in all patients (*P* > 0.05). Furthermore, there was no significant difference in the risk of cortical bone disruption between the Korean and Han patients regarding different impaction depths and root numbers (*P* > 0.05). When the impaction type was horizontal, the risk of cortical bone disruption in the Korean patients was 1.980 times higher than in the Han patients, which was statistically significant (*P* < 0.05).

## 4. Discussion

Mandibular third molar extraction is one of the most common surgical procedures in oral and maxillofacial surgery. When the mandibular canal is close to the MTM, an IAN injury is easily caused during MTM extraction, resulting in numbness and pain in the lower lip [18]. A safer approach is to use three-dimensional CBCT to obtain more information about the position of the neural tube, which can be used to determine the most appropriate therapeutic regimen.

### 4.1. General Clinical Data and Distribution of Impaction Types of Korean People from the Yanbian Area and the Han People

In this study, there were no significant differences in gender, age distribution, the number of MTMs in different genders, and the number of different sides between the Korean and Han people. This means that further statistical analysis could be performed. Chen et al. [19] found in a CBCT study of 1,304 patients that vertical impactions were the most common type of impaction, followed by mesial and horizontal impactions, and that all other types were rare. When the MTM intersected with the mandibular canal, Xu et al. [3] found in a CBCT study of 537 patients that mesial and vertical impactions were the most common type of impaction and that horizontal impactions were the least common. This study screened 260 Korean and Han patients whose MTM root tips were in contact with or overlapped the mandibular canal. It was found that mesial impactions were the most common, followed by horizontal impactions, vertical impactions, and finally, all other impaction types. The reason for this situation may be that the difficulty of vertical impaction extractions is low, so CBCTs are not taken. The patients selected in this study were those who had high-difficulty extractions for mesial and horizontal impactions.

### 4.2. Comparison of the Positional Relationship, Shape, and Cortical Bones of the Mandibular Canals and Mandibular Third Molars in Korean People from the Yanbian Area and the Han People

There are four positional relationships between the mandibular canal and MTM: subroot, buccal, lingual, and interroot. While it is difficult to show the relationship between the mandibular canal and the MTM in panoramic radiography, some studies believe that the mandibular canal is mainly located on the buccal side of the MTM [20]. However, this study found that the mandibular canal in both Korean and Han patients was mainly located below the root of the MTM, which is consistent with studies by Xu et al. [3] and Quirino de Almeida Barros et al. [21] Different regions, nationalities, and dietary habits lead to differences in craniomaxillofacial structures and tooth development [11]. Korean people are one of the main ethnic minorities in China, yet there are few studies comparing their MTM-mandibular canal positional relationships to those of the Han people. Therefore, in this study, the MTM-mandibular canal positional relationships between the Korean and Han people were studied. It was found that there were obvious nationality-based differences and that the differences were statistically significant (*P* < 0.05). Except for the position below the root, the buccal distribution rate of the Han patients was higher, which is consistent with the results of the studies by Xu et al. [3], and it was significantly larger than that of the Korean patients. The root-to-root incidence in Korean patients was higher than in the Han patients. The difference in the positional relationship affects the shape of the mandibular canal and the rate of cortical bone disruption, thus affecting preoperative assessments of IAN injuries and the selected surgical treatments.

Shiratori et al. [20] proposed that the cortical bone and the shape of the mandibular canal are reliable predictors of IAN injuries during MTM extractions. The dumbbell shape has a larger area of cortical bone disruption than the teardrop oval shape, and patients showing cortical bone loss and dumbbell shapes during MTM extraction should be considered high-risk patients for mandibular canal injury [7, 9, 20]. In terms of the rate of cortical bone disruption of the mandibular canal, the root and buccal side of the Korean and Han people is lower compared with the rate of cortical bone disruption between the root and the lingual side, which is consistent with the results of Chen et al. [19] and Maegawa et al. [17] There was no significant difference between the Korean and Han people for the disruption rates in either location.

Different positional relationships affect how force is applied during tooth extraction [22]. For example, when the mandibular canal is located on the lingual side, the tooth root presses the lingual bone plate to different degrees when the dental elevator is used for dislocation, causing IAN injury. Shujaat et al. [23] believed that there was a potential relationship between the impaction type and the positional relationship. It is worth noting that in the Saudi population, 71.6%–78.6% of the vertical, mesial, and horizontal impacted mandibular canals are located on the lingual side of the root. Here, there is a significant correlation between the impaction type and the positional relationship. However, Quirino de Almeida Barros et al. [21] did not find a correlation between the impaction type and the positional relationship in the Saudi population. The relationship between the impaction type and the positional relationship may be related to the ethnic group of the patient. In this study, the most commonly used clinical impaction types (vertical, mesial, and horizontal) were screened [3, 5, 19]. There was no correlation found between the impaction types and their locations. However, there was a difference in the positional relationship between the Korean and Han patients in the mesial and horizontal impactions. The proportion of mandibular canals between the roots for the Korean patients was significantly higher than that of the Han patients in mesial and horizontal impactions. In mesial impactions, the proportion occurring on the lingual side of the Han patients was higher than that of the Korean patients, which indicated that there were ethnic differences in the MTM-mandibular canal positional relationship under different impaction types.

This study explored the shortest distances in different positional relationships and cortical bone disruptions under different impaction types. It was found that the shortest distance measured in the Korean patients was smaller than that of the Han patients on the buccal side of the mesial and horizontal impactions. This means that Korean people are more likely to have cortical bone disruptions on the buccal side, which may be related to the thicker buccal bone plate of the Han people or that their nerve tubes are closer to the buccal cortical bone. Although the shortening of the distance does not directly become a risk factor for an IAN injury in clinical surgery, the disruption of the cortical bone usually exposes the mandibular canal during operation, thus becoming a risk factor for IAN injuries [6, 7, 24].

### 4.3. Study on the Rate of Cortical Bone Disruption in Korean People from the Yanbian Area and the Han People

In the MTM area, the mandibular canal has a complete bone tube composed of cancellous bone [25]. There is a continuous white line around the maxillary and mandibular canal on imaging [26], so it is called the cortical bone, with properties of cortical bone integrity and disruption. Cortical integrity is positively correlated with IAN injuries [6, 7], and studies have confirmed that exposed mandibular canals have a greater rate of IAN injury than unexposed mandibular canals [27]. The risk of cortical bone disruption was further analyzed using CBCT, which was compared with two-dimensional panoramic radiography [28].

Wang et al. [29] examined 136 patients using CBCT and found that the cortical bone interruption rate of the mandibular canal was 46.3%. Chen et al. [19] found that for 1,304 MTMs, the cortical bone interruption rate of the mandibular canal was 20.2%. Park et al. [6] found that in 259 MTMs, the cortical bone interruption rate of the mandibular canal was 36.7%. This study found that in 213 MTMs, the cortical bone interruption rate of the mandibular canal of the Han patients was 48.4%, which was similar to the results of Wang et al. [29] Jung et al. [30] found that in 175 MTMs in South Korea, the cortical bone interruption rate of the mandibular canal was 56%. In this study, it was found that in 208 MTMs of Korean patients, the cortical bone interruption rate of the mandibular canal was 55.8% higher than that of the Han patients. However, the ordered logistic regression analysis results found no significant difference in the risk of cortical bone disruption between the Korean and Han patients (*P* > 0.05).

After further classification of the impaction types, there was a significant difference in the risk of cortical bone disruption between the Korean and Han people. The risk of cortical bone disruption in the Korean patients was 1.980 times higher than that of the Han patients in horizontal impactions. This risk covered the calculation under the condition of intact noncontact cortical and intact cortical bones. It was more suitable for assessing the risk of injury to the IAN in clinical extractions of MTMs. The results showed that in the horizontal impactions, in terms of the mandibular canal interruptions in the interroot, the rate for the Korean patients was significantly higher than for the Han patients. Additionally, the mandibular canal interruption rate was the highest in the interroot for both the Korean and Han patients.

The deeper the impaction depth, the higher the risk of injury to the IAN of the MTM [31], as the deeper the teeth are, the more parts and opportunities there are to access the neural tube. Although the panoramic radiography screenings in this study have shown that the MTM root tip can come into contact with or overlap the mandibular canal, it was found that with the increase in depth, the cortical bone disruption rate also increases [19]. The average cortical bone disruption rate was as follows: high (47.4%), medium (52.5%), and low (70.8%) in the Korean and Han groups, but the increase in impaction depth was not significantly correlated with nationality.

Previous studies on MTMs rarely considered the root number [19] because the root numbers and morphologies of MTMs varied greatly. However, in clinical practice, compared with single-root MTMs, extracting MTMs with multiple roots has a greater probability of damage to the mandibular canal [32]. In this study, the number of MTM roots in the Korean patients was the largest in double-root teeth, followed by single-root teeth, which was consistent with the number of roots in Korean people [33]. In comparing the number of roots and the risk of cortical bone in the Han patients, no difference was found between the number of roots and the risk of cortical bone disruption between the Korean and Han patients. This also suggests that although an increase in the number of roots increases the risk of cortical bone disruption, it is not reflected in racial differences at the same number of roots.

This study also has some shortcomings; as it is a retrospective study, the statistical data and analysis results are affected by the population size, distribution, ethnic affiliation, and information errors in the region. Clarissa has reported the correlation of dental anomalies with certain patterns of malocclusion, an aspect that was not focused on in this study [34]. Additionally, future studies should be performed in order to evaluate also other variables and their possible relationship with third molar position, such as sella turcica bridging [35].

## 5. Conclusion

There are some differences in the positional relationship between the MTM and mandibular canal and the rate of cortical bone disruption between Korean people from the Yanbian area and the Han people, all of which should be paid attention to clinically.

## Figures and Tables

**Table 1:** Comparison of the age of Korean and Han Chinese by gender (P_50_ (P_25_–P_75_)).

	Korean nationality	Han nationality	*Z*	*P*
Male	30 (23–43)	27 (24–33)	−1.159	0.097
Female	27 (22–34)	26 (23–31)	−0.413	0.679

*Note*. *P* < 0.05 means statistical significance.

**Table 2:** Frequency analysis on Korean and Han nationalities by gender, location, and impaction type (*n* (%)).

	Korean nationality	Han nationality	*χ* ^2^/Fisher	*P*
Gender				
Male	105 (50.5)	100 (46.9)	0.526	0.468
Female	103 (49.5)	113 (53.1)		
Location				
Left side	106 (51.0)	106 (49.8)	0.060	0.806
Right side	102 (49.0)	107 (50.2)		
Impaction type				
Vertical	34 (16.3)	42 (19.7)	0.809	0.368
Mesial	94 (45.2)	80 (37.6)	2.529	0.112
Horizontal	70 (33.7)	80 (37.6)	0.700	0.403
Buccal	1 (0.5)	5 (2.3)	—	0.216
Buccolingual	2 (1.0)	2 (0.9)	—	1.000
Distal	2 (1.0)	0 (0)	—	0.244
Inversion	5 (2.4)	4 (1.9)	—	0.749

*Note*. *P* < 0.05 means statistical significance.

**Table 3:** Comparison between Korean and Han nationality in terms of the positional relationship between the mandibular canal and MTM and the rate of cortical bone disruption (*n* (%)).

	Korean nationality	Han nationality	*χ* ^2^	*P*
Positional relationship				
Below the root	107 (51.4)	94 (44.1)	2.254	0.133
Buccal side	43 (20.7)	70 (32.9)	7.964	0.005
Lingual side	28 (13.5)	38 (17.8)	1.526	0.217
Between roots	30 (14.4)	11 (5.2)	10.263	0.001
Cortical bone disruption rate				
Below the root	43 (40.2)	35 (37.2)	0.184	0.668
Buccal side	19 (44.2)	22 (31.4)	1.875	0.171
Lingual side	25 (89.3)	37 (97.4)	—	0.304
Between roots	29 (96.7)	9 (81.8)	—	0.170

*Note*. *P* < 0.05 means statistical significance.

**Table 4:** Comparison between Korean and Han nationality in terms of the relative positional relationship between the mandibular canal and MTM in different impaction types (*n* (%)).

	Korean nationality	Han nationality	*χ* ^2^/Fisher	*P*
Vertical				
Below the root	19 (55.9)	16 (38.1)	2.393	0.122
Buccal side	7 (20.6)	16 (38.1)	2.729	0.099
Lingual side	7 (20.6)	8 (19.0)	0.028	0.867
Between roots	1 (2.9)	2 (4.8)	—	1.000
Mesial				
Below the root	39 (41.5)	23 (28.7)	3.058	0.080
Buccal side	24 (25.5)	31 (38.8)	3.493	0.062
Lingual side	10 (10.6)	18 (22.5)	4.504	0.034
Between roots	21 (22.3)	8 (10.0)	4.739	0.029
Horizontal				
Below the root	43 (61.4)	48 (60.0)	0.032	0.858
Buccal side	11 (15.7)	21 (26.3)	2.469	0.116
Lingual side	8 (11.4)	10 (12.5)	0.041	0.840
Between roots	8 (11.4)	1 (1.3)	6.858	0.009

*Note*. *P* < 0.05 means statistical significance.

**Table 5:** Comparison between Korean and Han nationality in terms of the shortest distance between the mandibular canal and the root tip of MTM in different types of impaction (*n* (%)).

	Korean nationality	Han nationality	*Z*	*P*
Below the root				
Vertical	0 (0–1.940)	0.330 (0–2.640)	−0.632	0.527
Mesial	0 (0–1.830)	0 (0–0)	−1.581	0.114
Horizontal	0 (0–1.000)	0 (0–1.740)	−1.537	0.124
Buccal side				
Vertical	1.090 (0–4.350)	0 (0–1.915)	−0.685	0.493
Mesial	0 (0–0.370)	0 (0–2.150)	−2.029	0.042
Horizontal	0 (0–0)	1.090 (0–3.030)	−2.647	0.008

*Note*. *P* < 0.05 means statistical significance.

**Table 6:** Comparison between Korean and Han nationality in terms of relative position and shape of mandibular canal and MTM (*n* (%)).

		Round/oval	Teardrop shape	Dumbbell shape	*χ* ^2^/Fisher	*P*
Below the root	Korean	76 (71.0)	25 (23.4)	6 (5.6)	1.784	0.410
	Han	74 (78.7)	15 (16)	5 (5.3)		

Buccal side	Korean	20 (46.5)	13 (30.2)	10 (23.3)	3.507	0.173
	Han	45 (64.3)	15 (21.4)	10 (14.3)		

Lingual side	Korean	5 (17.9)	1 (3.6)	22 (78.6)	4.467	0.113
	Han	4 (10.5)	8 (21.1)	26 (68.4)		

Between roots	Korean	7 (23.3)	1 (3.4)	22 (73.3)	1.955	0.362
	Han	4 (36.4)	1 (9.1)	6 (54.5)		

*Note*. *P* < 0.05 means statistical significance.

**Table 7:** Ordered logistic regression analysis results of different nationalities, impaction type, depth, number of roots, and cortical bone state.

Features			Cortical bone state				
			Cortical bone is intact without contact	Cortical bone is intact with contact	Cortical bone disruption		

Nationality		Han	69	41	103		1
		Korean	53	39	116	0.094	1.366 (0.948–1.967)

Impaction type	Vertical	Han	16	11	15		1
		Korean	13	6	15	0.678	1.194 (0.516–2.766)
	Mesial	Han	19	15	46		1
		Korean	22	16	56	0.824	1.069 (0.596–1.917)
	Horizontal	Han	33	11	36		1
		Korean	14	16	40	0.031	1.980 (1.066–3.676)

Impaction depth	High position	Han	40	15	50		1
		Korean	35	21	50	0.744	1.088 (0.654–1.811)
	Median position	Han	27	22	41		1
		Korean	16	12	44	0.068	1.749 (0.960–3.188)
	Low position	Han	2	4	12		1
		Korean	2	6	22	0.588	1.413 (0.404–4.945)

Number of roots	Single root	Han	11	10	13		1
		Korean	9	6	22	0.127	1.997 (0.821–4.857)
	Double roots	Han	54	30	82		1
		Korean	42	30	77	0.531	1.143 (0.752–1.739)
	Three roots	Han	4	1	8		1
		Korean	2	2	16	0.198	2.761 (0.589–12.944)
	Four roots	Han	0	0	0		
		Korean	0	1	1		1

*Note*. *P* < 0.05 means statistical significance.

## Data Availability

The datasets used and/or analysed during the current study are available from the corresponding author on reasonable request. The authors declared that materials described in the manuscript, including all relevant raw data, will be freely available to any scientist wishing to use them for noncommercial purposes, without breaching participant confidentiality.
